# Are we underestimating the genetic variances of dimorphic traits?

**DOI:** 10.1002/ece3.1361

**Published:** 2015-01-08

**Authors:** Matthew E Wolak, Derek A Roff, Daphne J Fairbairn

**Affiliations:** Department of Biology and Graduate Program in Evolution, Ecology, and Organismal Biology, University of CaliforniaRiverside, California, 92521

**Keywords:** Animal model, between-sex genetic correlation, offspring-parent covariance, sexual dimorphism, sire and dam variances

## Abstract

Populations often contain discrete classes or morphs (e.g., sexual dimorphisms, wing dimorphisms, trophic dimorphisms) characterized by distinct patterns of trait expression. In quantitative genetic analyses, the different morphs can be considered as different environments within which traits are expressed. Genetic variances and covariances can then be estimated independently for each morph or in a combined analysis. In the latter case, morphs can be considered as separate environments in a bivariate analysis or entered as fixed effects in a univariate analysis. Although a common approach, we demonstrate that the latter produces downwardly biased estimates of additive genetic variance and heritability unless the quantitative genetic architecture of the traits concerned is perfectly correlated between the morphs. This result is derived for four widely used quantitative genetic variance partitioning methods. Given that theory predicts the evolution of genotype-by-environment (morph) interactions as a consequence of selection favoring different trait combinations in each morph, we argue that perfect correlations between the genetic architectures of the different morphs are unlikely. A sampling of the recent literature indicates that the majority of researchers studying traits expressed in different morphs recognize this and do estimate morph-specific quantitative genetic architecture. However, ca. 16% of the studies in our sample utilized only univariate, fixed-effects models. We caution against this approach and recommend that it be used only if supported by evidence that the genetic architectures of the different morphs do not differ.

## Introduction

The fundamentals of quantitative genetics (Fisher 1918) provide the theoretical foundation for most of evolutionary ecology (Kruuk et al. 2008) and the adoption of quantitative genetic methods in evolutionary ecology research enables us to make quantitative predictions about the rate and direction of phenotypic evolution (Wilson et al. 2010). The central paradigm in evolutionary quantitative genetics is to partition phenotypic variation into contributions from additive genetic as well as nonadditive genetic and environmental variances (Roff 2006). Although recent work has highlighted other contributions to phenotypic variance, such as common environment, maternal genetic, and spatial autocorrelation among relatives (Kruuk et al. 2001; MacColl and Hatchwell 2003; Charmantier et al. 2004; Wilson et al. 2005; Kruuk and Hadfield 2007; Stopher et al. 2012), estimates of additive genetic variance are of paramount importance for predicting population responses to natural selection (Kruuk et al. 2008) using the breeder's equation (Falconer 1989; Lynch and Walsh 1998) or the Secondary Theorem of Natural Selection (Robertson 1966; Price 1970). However, several recent critiques have argued that researchers in evolutionary ecology often improperly implement and interpret quantitative genetic techniques (Wilson 2008; Hadfield et al. 2010). These critiques stress the need for a deeper understanding of the basic theory underlying quantitative genetic techniques for partitioning phenotypic variance and suggest that such an understanding is a prerequisite for progress in our study of evolutionary processes (Postma 2006; Wilson 2008; Hadfield et al. 2010). In this study, we address a common problem associated with estimating additive genetic variances and covariances for polygenic traits that are expressed as discrete morphs.

Additive genetic variances are defined within the context of a specific population and environment. However, traits are often expressed in two different environments or phenotypic classes within a single population. This is true of dimorphic traits such as disease incidence, wing dimorphisms, protective dimorphisms, trophic dimorphisms, mating dimorphisms, and life cycle dimorphisms (reviewed in Roff 1996) and also of traits differing between the sexes, for example, sexual dimorphisms in behavior, morphology, physiology, and life history (reviewed in Fairbairn et al. 2007; Fairbairn 2013). Morph can be considered as an environment which interacts with genes to alter the average genetic effects. An individual's average genetic effect for a polygenic trait in a population is known as its breeding value. Because breeding values are expressed as an individual's average deviation from the population mean, the mean breeding value equals zero and the variance in breeding values is the additive genetic variance (Falconer 1989). Polygenic traits occurring in both phenotypic classes (morphs) are often exposed to different selective environments within each morph, leading to selection for different average allelic effects in each morph and ultimately to the evolution of morph-specific genetic effects that can be modeled as genotype-by-environment interactions (Roff 1997). If there are only two morphs (environments), the genotype-by-morph interaction can be expressed as a genetic correlation between morphs (Falconer 1952). Similarly, the set of genetic correlations can be described for instances when there are more than two morphs (environments). The latter may occur when, for example, traits are expressed across life-history stages. Overall, the genetic correlation between morphs summarizes the relationship between the ranks of breeding values expressed in one morph relative to the rank in the other.

Evolutionary ecologists making statistical inferences on breeding values commonly treat dimorphic variation by including the morph as a fixed effect in statistical models to remove the average difference between the morphs (e.g., Table S2.1 in Appendix S2; Wilson et al. 2010; Roff and Fairbairn 2011; see also WAMWiki at http://www.wildanimalmodels.org/tiki-index.php). Although this is necessary to control for fixed differences in phenotypic means between the morphs, it does not affect the correlation between morphs in breeding values (i.e., between-morph additive genetic correlation). By itself, using a fixed effect of morph invokes the biological assumption of a perfect additive genetic correlation between the two morphs. Although the statistical implications of this assumption are generally understood, neither in the quantitative genetic literature nor the evolutionary ecology literature have the effects of this assumption on quantitative genetic parameter estimates been formally quantified and thoroughly explained. Consequently, key quantitative genetic parameters in evolutionary ecology are not being estimated (e.g., additive genetic correlation) or are potentially being miscalculated (e.g., additive genetic variance). In this study, we describe the bias in estimates of additive genetic variance that arises when the additive genetic effects of a trait are assumed to be perfectly correlated between two morphs. As expected (Roff and Fairbairn 2011), we find that whenever the between-morph additive genetic correlation is less than one, the additive genetic variance for the morphs combined will be underestimated when only a fixed effect of morph is specified. We show how to estimate the magnitude of this bias for a variety of quantitative genetic variance partitioning methods employed by evolutionary ecologists (e.g., offspring-parent regression, half-sib ANOVA, and mixed effect models of pedigreed populations).

## Predicted Bias in Estimates of Quantitative Genetic Parameters for Dimorphic Traits

In practice, breeding values are estimated using a combination of phenotypic information and the relatedness among individuals within a population. Although an individual can never be simultaneously measured for both phenotypes in a dimorphism, each individual carries genes that will contribute to both phenotypes. Therefore, breeding values for phenotypes that are never expressed can still be measured. A common example of this is milk production in dairy cattle, where bulls cannot be measured for milk yield (e.g., Mrode 2005). However, bull breeding values for milk yield can be estimated for the purposes of determining which bulls will produce daughters with the highest milk yield. Information for the bull's breeding value is gathered from female relatives that share some proportion of genes that the bull carries for the milk yield trait.

In a hypothetical population, if every individual mates with every other individual and offspring are produced from each mating, then breeding values can be estimated as two times the deviation of an individual's average offspring phenotype from the population mean phenotype (Lynch and Walsh 1998, p. 73). This concept of breeding value is useful for examining the effect of genotype-by-morph interactions on the distribution of breeding values for each morph within a population. If the average genetic effect of an allele differs between morphs, the breeding values of the two morphs will also differ. For example, consider height in an imaginary population of dimorphic organisms. A genotype's breeding value for height in morph M1 is defined as the average genetic effect of its genes on height when expressed in morph M1. The breeding value for the same genotype in morph M2 is defined as the average genetic effect of its genes on height when expressed in morph M2. For this one genotype, breeding values are estimated as two times the deviation of the average phenotype of morph M1 offspring from the morph M1 population mean and similarly for morph M2.

**Figure 1:**
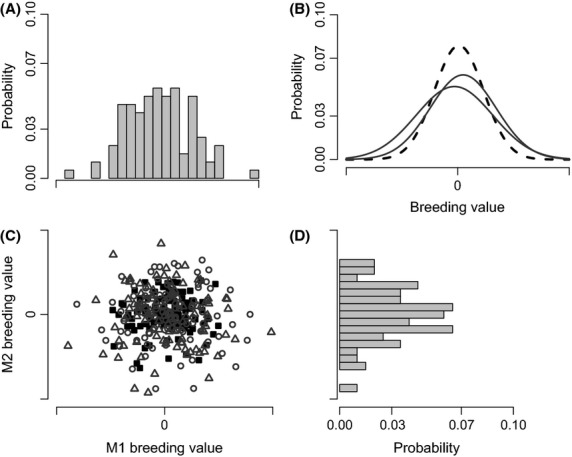
A single, simulated distribution of breeding values when the variances for morphs M1 and M2 equal 50 and the between-morph correlation equals zero. (A) the probability distribution of breeding values for morph M1, (B) the probability distributions of the average breeding values (black dashed line) and those for morph M1 and M2 (black solid lines), (C) a scatter plot of the average breeding values (black filled squares) and the breeding values for morphs M1 (open triangles) and M2 (open circles), and (D) the probability distribution of breeding values for morph M2.

The distribution of breeding values (**a**_**1,**_ **a**_**2**_) for the trait in the two morphs of a population can be described by a bivariate normal distribution, where each has a mean of zero, a variance according to the morph-specific variance in trait breeding values [*Var*(**a**_**1**_) and *Var*(**a**_**2**_)], and some correlation between breeding values in the two morphs (i.e., *r*_**a-1,2**_; Fig. 1). When the effect of morph on average genetic effects is ignored, the breeding values for a trait are defined as the average genetic effects when a genotype is expressed in both morph M1 and morph M2 (i.e., the average of morph M1 and M2 breeding values). The distribution of these average breeding values (**a**_**u**_) can be described by a univariate normal distribution with a mean of zero and variance equal to the variance in average breeding values, *Var*(**a**_**u**_) (Fig. 1). The variance in **a**_**u**_ can be predicted from the general formula for the variance of two random variables averaged together: 

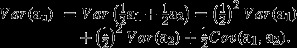
1

Illustrations of the morph M1, morph M2, and average breeding value distributions, using one set of random draws from each respective distribution, are shown in Figure1. When the morphs have the same additive genetic variance [*Var*(**a**_**1**_)=*Var*(**a**_**2**_)], an algebraic rearrangement of equation 1 shows that the variance in average breeding values, *Var*(**a**_**u**_), will be less than both *Var*(**a**_**1**_) and *Var*(**a**_**2**_) whenever the between-morph additive genetic correlation is less than unity. This is seen in Figure1, where the spread of points is greater for the breeding values of morphs M1 and M2 than it is for the spread in average breeding values (Fig. 1C), and the probability distributions for breeding values of morphs M1 and M2 are wider than the probability distribution of the average breeding values (Fig. 1B).

Assuming that *Var*(**a**_**1**_)≥*Var*(**a**_**2**_), a rearrangement of the right hand side of equation 1 shows that the variance in average breeding values, *Var*(**a**_**u**_), will be less than either of the two morphs’ additive variances whenever

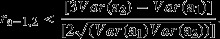
2

This downward bias is illustrated in Figure[Fig fig02], for the range of possible between-morph genetic correlations when *Var*(**a**_**2**_) is 10% less than *Var*(**a**_**1**_). See Appendix  1 for the analogous equation predicting the between-morph genetic correlation at which the heritability of the morphs combined will be less than either of the morph-specific heritabilities.

**Figure 2 fig02:**
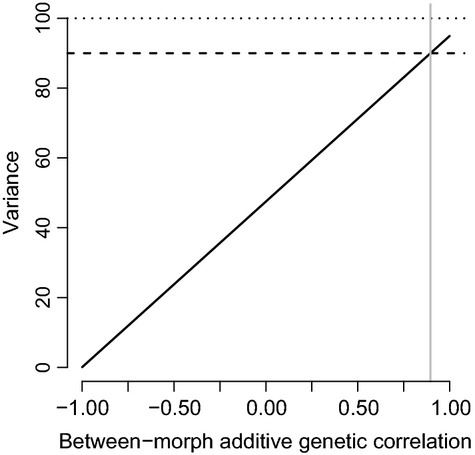
The predicted average variance (solid-black, diagonal line) when morph M1 variance equals 100 (dotted-black, horizontal line) and morph M2 variance equals 90 (dashed-black, horizontal line) using equation 1 from the text. The average variance will be less than either of the two variances whenever the between-morph additive genetic correlation is less than approximately 0.89 (vertical-grey line; see equation 2 in text). The variance on the *y*-axis can either be the additive genetic variance, offspring-parent covariance, sire variance, or dam variance.

In practice, true breeding values are unknown, and thus, the additive genetic variance must be estimated using known contributions of additive variance to the phenotypic resemblance between relatives. For many breeding designs, the additive genetic variance is estimated as a fraction of the covariance between offspring and parent phenotypes or as a fraction of the sire or dam variance components (Falconer 1989). Alternatively, additive genetic variance estimates can be obtained from mixed effect statistical models which simultaneously consider all pairwise relationships. This latter approach enables estimation of additive genetic variance in nonstandard breeding designs and wild populations for which a population pedigree is available. Below, we consider each approach separately.

### Offspring-parent and half-sib models

Methods of estimating the additive genetic variance (i.e., the variance in breeding values) from the covariance between offspring and parents, or the variance among half-sib families also depend on there being no genotype-by-morph interaction for breeding values expressed in two different morphs (in addition to other assumptions regarding random mating, nonadditive genetic effects, and inbreeding; Falconer 1989; Lynch and Walsh 1998). If these assumptions hold, the additive genetic variance equals two times the covariance between offspring and parent phenotypes and four times the variance among sire or dam family (nested within sire) phenotypes in a nested half-sib breeding design (Falconer 1989; Lynch and Walsh 1998). When equation 1 is multiplied by ½ or ¼, it also predicts the offspring-parent covariance or the sire/dam variance, respectively. Figure2 can be interpreted as depicting the line that predicts either the joint offspring-parent covariance or joint sire/dam variances over a range of between-morph additive genetic correlations when the (co)variance in one morph is 10% less than the other. For example, when the two morphs are two sexes, equation 2 predicts that the mid-offspring on mid-parent covariance will be less than the sire on male offspring covariance of 90 when the female offspring on dam covariance is 100 and the between-sex additive genetic correlation is approximately 0.89 (Fig. 2, grey vertical line).

Morph-specific offspring-parent regressions or nested linear models are therefore necessary when the between-morph additive genetic correlation is less than one or the additive genetic variances differ between the morphs. Bivariate statistical models, where the phenotypes in the two morphs are treated as separate traits, can also be utilized to obtain morph-specific observed (co)variance components. The additive genetic (co)variances can then be estimated from sire, dam, and within-family (co)variances (e.g., Cowley et al. 1986).

### Animal models

The range of organisms and populations for which researchers can obtain predictions of breeding values and make inferences about the additive genetic variance in populations has broadened with the adoption of the mixed effects linear model commonly known as the “animal model” (Henderson 1973; Lynch and Walsh 1998; Kruuk 2004). Animal models have become popular tools in evolutionary ecology because of their potential to disentangle confounding sources of similarity between relatives, simultaneously consider relationships beyond offspring-parent or half- and full-siblings in the estimation of variance components and obtain unbiased estimates of model parameters when selection has occurred during a given study (Lynch and Walsh 1998; Kruuk 2004).

Here, we consider the effect of the between-morph additive genetic correlation on joint estimates of variance components in animal models. Estimating one additive variance for both morphs in an animal model assumes no genotype-by-morph interactions and, therefore, a between-morph additive genetic correlation of one. A univariate analysis incorporating these assumptions models the phenotypic observations **y**, as a function of breeding values, **a** (details in Appendix S1, *Univariate model*). The breeding values in **a** are assumed normally distributed with mean of zero and variance of *Var*(**a**) = **G**_**a**_⊗**A**, where **A** is the additive genetic relationship matrix (⊗ symbolizes the direct product between two matrices). In this model, **G**_**a**_ = *σ*^**2**^_**a**_ where *σ*^**2**^_**a**_ is the additive genetic variance in the base population. Thus, the assumption regarding the relationship between morph M1 and M2 breeding values for the univariate model (see equation S1.1 in Appendix S1) is that all breeding values are modeled from a univariate distribution of random effects.

Alternatively, the phenotype of interest can be modeled as a different trait for each morph (e.g., Mrode 2005, p. 106) by estimating morph-specific variances. This approach is analogous to estimating additive genetic variance in two environments (Roff 1997; Roff and Fairbairn 2011). In practice, this is carried out by specifying a bivariate model where the two traits modeled represent the phenotype as expressed in morph M1 and morph M2 (details in Appendix S1, *Bivariate model*). In such a model, only morph M1 traits are expressed in morph M1 and only morph M2 traits are expressed in morph M2. Accordingly, all morph M1 individuals will have missing phenotypes for the morph M2 trait and vice versa for morph M2 individuals. The intercept, or overall mean, of the model accounts for a difference in the means of unstandardized phenotypes for morphs M1 and M2. This approach makes a residual covariance between the two morphs impossible to define as no individual can express the trait in both morphs (i.e., morph M1 phenotypes cannot be expressed in morph M2; e.g., Mrode 2005). When the data permit, the bivariate description is preferred over separate univariate models for each morph, because it allows for estimation of the between-morph genetic correlation and increases the precision with which BLUPs for the breeding values are obtained. The latter point arises from the additional information used to determine the breeding values for one morph derived from the expression of the phenotype in opposite morph relatives (analogous to the above example where one can estimate a bull's milk yield breeding value; Mrode 2005).

In the bivariate model (see equation S1.2 in Appendix S1), the breeding values in **a** (the bivariate distribution of **a**_**1**_ and **a**_**2**_) are multivariate normally distributed. Consequently, *Var*(**a**) = **G**_**a**_⊗**A**, but here **G**_**a**_ is the 2 × 2 matrix: 

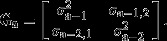
3

When a univariate animal model only includes morph as a fixed effect, the separate distributions of breeding values for the two morphs are assumed perfectly correlated (i.e., *r*_**a-1,2**_ = 1). Thus, **G**_**a**_ in equation 3 is forced to satisfy *σ*^**2**^_**a-1**_=*σ*^**2**^_**a-2**_=*σ*_**a-1,2**_ (*r*_**a-1,2**_=1 when this occurs), and the bivariate model (equation S1.2) is equivalent to the univariate model (equation S1.1). When these assumptions are valid (i.e., *σ*^**2**^_**a–1**_ = *σ*^**2**^_**a–2**_ = *σ*_**a-1,2**_), mixed effect models treating any differences between the morphs as a fixed difference (i.e., morph as a fixed effect and jointly modeling the morphs) will produce unbiased estimates of the additive genetic variance in the population. However, if the between-morph additive genetic correlation is less than unity (*r*_**a–1,2**_ ≠ 1) as illustrated in Figure1, the univariate model (equation S1.1 in Appendix S1) will produce a biased estimate of additive genetic variance as predicted by equation 2 (Fig. 2).

## Examples from the Literature

Results from many empirical papers can be interpreted in the context of the dynamics described by equations 1 and 2. For example, in a study of the genetic basis of life-history trade-offs Roff and Fairbairn (2011) analyzed five traits in two wing morphs of the cricket, *Gryllus firmus*. They initially estimated heritability when the sexes or wing morphs were combined followed by analyses where sex and wing-morph-specific heritabilities were estimated. For a number of traits, the combined estimates of heritability were lower than the sex-specific or wing-morph-specific estimates. They postulated that these traits had between-sex or between-morph genetic correlations less than one. In agreement with their predictions, and our predictive equations, Roff and Fairbairn (2011) confirmed the presence of genetic correlations between-sexes or wing-morphs significantly less than one in the same traits where they found the combined heritability estimate was lower than the sex-specific or wing-morph-specific estimates. Roff and Fairbairn's results highlight that morphs within a population, particularly the two sexes, often have different distributions of breeding values that reflect the different evolutionary processes (e.g., selection) experienced by the morphs.

The proposal that it is necessary to consider the quantitative genetic architecture of a trait separately for each sex is not new (e.g., Fedorka et al. 2007). In agricultural breeding, the approach has often been to compare differences in parameter estimates from models that do or do not consider the sexes separately (e.g., Garrick et al. 1989; Rodríguez-Almeida et al. 1995; Lee and Pollak 1997; Van Vleck and Cundiff 1998; Näsholm 2004). Careful consideration of results from these studies demonstrates that the sex-specific and combined-sex additive genetic variance estimates differ greatly when the between-sex genetic correlations are significantly less than unity. Consequently, recommendations as to the separate or combined consideration of the sexes are proffered on a study by study basis.

Taken together, empirical support for separate estimates of morph-specific additive genetic variances (e.g., Roff and Fairbairn 2011) and evolutionary theory both promote adoption of a null model that considers the quantitative genetics of discrete morphs as different. To determine how often this occurs in practice, we sampled the recent literature and noted how researchers have treated discrete morphs in quantitative genetic analyses (details in the Appendix S2). We included papers estimating genetic variances or heritabilities that were published in *Evolution*,*the American Naturalist*,*Journal of Evolutionary Biology*, and *Heredity* between January 2013 and October 2014 (Table S2.1 in Appendix S2).

The most common taxa in these papers are insects (31 papers), birds (21 papers), and plants (11 papers). We found 79 papers estimating quantitative genetic parameters, 63 of which studied traits expressed by discrete morphs or classes within a population or breeding design. Sex was the most common dimorphic trait (35 of 63), but discrete environments, resource availabilities (e.g., diet treatments), ages, or populations/lines were also common. Note, however, that morph classifications are not mutually exclusive. For example, Ingleby et al. (2013) studied cuticular hydrocarbons in *Drosophila simulans* that were measured on individuals in separate diet and temperature treatments as well as in both sexes.

Only one of the 63 studies ignored the effects of the discrete morphs in their data. Of the studies that explicitly considered dimorphism (or polymorphism) in their analyses, 53 included a fixed effect (see Appendix S2), 44 estimated morph-specific additive genetic variances or heritabilities within the same model (e.g., the bivariate model in equation S1.2 Appendix S1) and just over one-third (24 of 62) analyzed morphs in separate models. In many cases (12), researchers used more than one method, for example, a univariate, fixed-effects model or a bivariate model combined with separate analyses of the two morphs. However, in 10 studies, the dimorphic or polymorphic variation was analyzed only using a fixed effects, univariate model (equation S1.1 in Appendix S1). The methods used to analyze data in these 10 studies were animal models (8), full-sib variance partitioning model (1), and parent-offspring regression (1).

In the absence of supplemental models or previous estimates from the study populations, it is not possible to determine whether the additive genetic variance estimates are biased downward in the 10 studies that relied only on univariate models (e.g., as predicted by equation 2). In some cases, univariate models are sufficient, but oftentimes not. Examples from studies that fitted both the univariate and bivariate models illustrate this point. Weiß and Foerster (2013) studied dominance rank in greylag geese (*Anser anser*) and found that the sex-specific heritability estimates (i.e., from the bivariate model, equation S2.2 in Appendix S2) were much higher than the estimate when the sexes were combined (i.e., from the univariate model, equation S2.1 in Appendix S2). As expected from our equation 2, their result can be explained in part by the low between-sex correlation (see table 3 in Weiß and Foerster 2013). Conversely, Berger et al. (2013) found no differences between additive genetic variances estimated for development rate in *Sepsis punctum* in two food treatments and thus combined these two classes for further analyses. In a third example, Schaper et al. (2013) were unable to fit a model of great tit (*Parus major*) gonadal size across months given the dataset available. Instead, they allowed for month-specific additive genetic variances by fitting separate models for each month. These studies illustrate the recommended practice of including dimorphic (or polymorphic) trait variation in analyses and only simplifying the models after confirming similar morph-specific additive genetic variances and between-morph additive genetic correlations of approximately one.

## Discussion

Estimates of additive genetic variance are at the heart of many studies in evolutionary ecology that are conducted to answer general questions regarding (1) the evolutionary forces that shape additive variance; and (2) population responses to selection. Discrete morphs or phenotypic classes occur in some species by virtue of different patterns of gene expression. There is no a priori reason to assume that the patterns of variances within and covariances among traits should be the same for traits expressed in these two genetic environments. Therefore, initial estimates of variances and covariances should consider the separate morphs or classes as distinct with the potential for genotype-by-morph interactions between them. As demonstrated above, when such genotype-by-morph interactions are not explicitly considered the resulting variance in the joint distribution of breeding values will be less than the variance in breeding values for either class. Thus, the estimated effect of evolutionary forces on additive genetic variance and predictions for evolutionary responses in mean phenotype may differ substantially based on the way phenotypes are modeled in a quantitative genetic analysis (i.e., joint distribution of breeding values versus morph-specific distributions). The common practice from papers in our literature sample (ca. 84%) and the approach we argue for here is to estimate morph-specific additive genetic variances and only combine traits across morphs when there is no evidence for morph-specific genetic architecture (i.e., when morph 1 and 2 (co)variances are *σ*^**2**^_**a–1**_ = *σ*^**2**^_**a–2**_ = *σ*_**a-1,2**_).

Downward biases in estimates of additive genetic variance exacerbate problems with quantitative genetic inference in limited datasets (e.g., some wild populations). Such datasets often do not have the sample size or informative relationships necessary to disentangle additive variance from other sources of phenotypic resemblance among relatives (e.g., see discussion above of Schaper et al. 2013). These issues are compounded when the statistical model attributes less of the phenotypic variance to additive genetic effects then it should because of the biases discussed above. The extent of this problem will, in part, be dictated by the additive genetic correlation between the trait values in the morphs.

Although the arguments made above have been framed within a single trait context for simplicity and ease of interpretation, the results extend to multivariate trait relationships where the pattern of covariances among traits will often differ between classes as well. This point is particularly salient for predicting evolutionary change using the statistical relationship between breeding values of a trait and of relative fitness (Secondary Theorem of Natural Selection; Robertson 1966; Price 1970). For example, studies of sexually dimorphic traits often find differences between the sexes in among-trait covariance matrices (e.g., Preziosi and Roff 1998; Jensen et al. 2003; Fedorka et al. 2007; Steven et al. 2007; Walling et al. 2008; Roff and Fairbairn 2011) which have been shown to impact predicted responses to selection (Fedorka et al. 2007).

## Appendix 1

Narrow-sense heritability, the ratio of the additive genetic variance to the phenotypic variance, is central to predicting the amount by which the average phenotype in a population will change from one generation to the next using the breeder's equation (Falconer 1989). Assuming equal residual variances for both morphs, if the heritability in morph M1 equals the heritability in morph M2, equation 1 can be extended to show that the heritability of the morphs combined will underestimate the within-morph heritabilities whenever the between-morph additive genetic correlation is less than one (Roff and Fairbairn 2011). If the heritabilities in morphs M1 and M2 are not equal, then by a similar rearrangement to the one which produces equation 2, the between-morph genetic correlation at which the heritability of the morphs combined will be less than both of the heritabilities in the two morphs occurs when

A1

In equation A1, **p**_**1**_ and **p**_**2**_ refer to the distributions of M1 and M2 phenotypes, respectively.

### Supporting Information

**Appendix S1.** Univariate and bivariate animal models. **Appendix S2.** Literature search.
