# Supplementary material for: Are we underestimating the genetic variances of dimorphic traits?
Source: Ecol Evol. 2015 Jan 8;5(3):590–7. doi: 10.1002/ece3.1361 (PMC4328764; doi:10.1002/ece3.1361)
Supplement: Supplementary file 1 [file ece30005-0590-sd1.pdf]

Supporting Information

ARE WE UNDERESTIMATING THE GENETIC VARIANCES OF DIMORPHIC TRAITS?

Wolak, M.E.*, D.A. Roff, and D.J. Fairbairn

*Corresponding author: Matthew Wolak; Address: School of Biological Sciences, Zoology Building, University of Aberdeen, Tillydrone Avenue, Aberdeen AB24 2TZ, UK. E-mail: matthew.wolak@abdn.ac.uk. Phone: +44 1224 273255.

S1 Univariate and bivariate animal models

Univariate model

A univariate animal model of a trait expressed in two morphs within the population can be specified as:

$$\mathbf{y} = \mathbf{X}\boldsymbol{\beta} + \mathbf{Z}_{\mathbf{a}}\mathbf{a} + \mathbf{e} \qquad \text{(S1.1)}$$

When every individual has only one measurement, **y** is a nx1 vector of phenotypes in a population of n individuals and **X** is a nxf design matrix (i.e., contains 0s and 1s), where f is the number of levels of fixed effects in the model, and contains 1s in rows occupied by observations with a particular level of the fixed effect in the fth column. The matrix **X** relates the observation in **y** to the appropriate fixed effect (mean) in $\boldsymbol{\beta}$. If morph is included as a fixed effect to account for differences in mean phenotype, then **X** contains 1s in the first column at rows occupied by morph M1 (corresponding to rows in **y**) and 1s in the second column at rows occupied by morph M2. The matrix $\mathbf{Z}_{\mathbf{a}}$ is an nxn design matrix which associates the phenotypic observation in **y** to the breeding value in **a**. The variables **a** and **e** are the nx1 vectors of additive genetic effects and environmental effects, respectively. The random variables **a**

and **e** are assumed normally distributed with means of zero and variances of $Var(\mathbf{a})=\mathbf{G_a}\otimes\mathbf{A}$, where **A** is the additive genetic relationship matrix ($\otimes$ symbolizes the direct product between two matrices), and $Var(\mathbf{e})=\mathbf{R}\otimes\mathbf{I}$, where **I** is an identity matrix (nxn, with 1s along the diagonal). In this model, $\mathbf{G_a}=\sigma^2_{\mathbf{a}}$ where $\sigma^2_{\mathbf{a}}$ is the additive genetic variance in the base population and $\mathbf{R}=\sigma^2_{\mathbf{e}}$, the environmental variance.

Bivariate model

A bivariate model of a trait expressed in two morphs within the population can be specified as:

$$\begin{bmatrix}\mathbf{y}_1\\ \mathbf{y}_2\end{bmatrix}=\begin{bmatrix}\mathbf{X}_1 & \mathbf{0}\\ \mathbf{0} & \mathbf{X}_2\end{bmatrix}\begin{bmatrix}\beta_1\\ \beta_2\end{bmatrix}+\begin{bmatrix}\mathbf{Z}_{\mathbf{a}1} & \mathbf{0}\\ \mathbf{0} & \mathbf{Z}_{\mathbf{a}2}\end{bmatrix}\begin{bmatrix}\mathbf{a}_1\\ \mathbf{a}_2\end{bmatrix}+\begin{bmatrix}\mathbf{e}_1\\ \mathbf{e}_2\end{bmatrix} \tag{S1.2}$$

In equation S1.2, **a** (the bivariate distribution of $\mathbf{a}_1$ and $\mathbf{a}_2$) and **e** (the bivariate distribution of $\mathbf{e}_1$ and $\mathbf{e}_2$) are assumed to represent random effects described by multivariate normal distributions. The $Var(\mathbf{a})=\mathbf{G_a}\otimes\mathbf{A}$, where $\mathbf{G_a}$ is a 2x2 matrix (see equation 3 in main text).

S2 Literature search

To evaluate the extent to which morph-specific estimates of additive genetic variances are estimated in the literature, we sampled four journals that present the majority of estimates of additive genetic variances in evolutionary ecology: *Evolution*, *the American Naturalist*, *Journal of Evolutionary Biology*, and *Heredity*. For each journal we included the issues for January through December 2013 and January through October 2014 (22 months). We read the title, abstract, and keywords of every paper published in this sample to determine if genetic variances or heritabilities were estimated. If yes, we read the full paper and categorized it according to: (1) the type of trait studied (monomorphic, dimorphic, or polymorphic), (2) whether morph was included in the statistical analysis, (3) whether morph was included as a fixed effect, (4) whether a bivariate model of both morphs (or multivariate in the case of a polymorphism) was used to estimate morph-specific genetic variances, and (5) whether morphs were analyzed in separate statistical models (regardless of how the study was categorized in 4).

We considered morph was included as a fixed effect (3) if it was either entered directly in the statistical model as a fixed effect or if the raw phenotypic data were mean centered across morphs before analyses (effectively the same as including fixed effect in the model). Similarly, we considered the morph to be modeled using a bivariate model (4) if the morphs were entered as separate traits or if a random interaction with morph was included (e.g., morph-by-sire family in a sire variance component model or morph-by-identity in an animal model). Finally, we did not include papers that either estimated additive genetic variances and heritabilities from simulated data or estimated mutational genetic variances.

Table S2.1 Results from the literature sample

Ref.	Organism	Trait(s)	1-Morph	2-Morph in analysis	3-Fixed effect	4-Bivariate model	5-Separate morph models
1	Hyla versicolor	advertisement call	Dimorphic	yes	yes	no	no
2	Agalychnis callidryas	risk-induced hatching timing & morphology	Dimorphic	yes	yes	yes	no
3	Tyto alba	color spots	Dimorphic	yes	yes	no	no
4	Tyto alba	nestling vocalizations	Dimorphic	yes	yes	no	no
5	Hirundo rustica	flight initiation distance	Polymorhpic	no	no	no	no
6	Cyanistes caeruleus	egg characteristics	Polymorphic	yes	yes	yes	no
7	Cyanistes caeruleus	body mass	Polymorphic	yes	yes	yes	no
8	Ficedula albicolis	tail, tarsus, wing, & mass	Dimorphic	yes	yes	no	no
9	Ficedula albicollis and Taeniopygia guttata	morphology & sexually selected traits	Dimorphic	yes	yes	no	no
10	Falco tinnunculus	coloration, body mass, & immunity	Dimorphic & Polymorphic	yes	yes	no	no (age) yes (sex)
11	Acrocephalus arundinaceus	wing tarsus length	Dimorphic	yes	yes	yes	yes
12	Parus major	oxidative damage & resistance to oxidative stress	Dimorphic	yes	yes	yes	yes
13	Parus major	clutch size	Monomorphic	NA	NA	NA	NA
14	Parus major	gonad size	Polymorphic	yes	yes	yes	yes
15	Parus major	egg-laying dates (female)	Dimorphic	yes	yes	yes	no
16	Parus major and Taeniopygia guttata	plumage & beak color	Dimorphic	yes	yes	no	no
17	Anser anser	dominance rank & aggression	Dimorphic	yes	yes	yes	yes
18	Perisoreus infaustus	body mass	Dimorphic	yes	yes	no	no
19	Melospiza melodia	female extra-pair reproduction and male paternity success	Monomorphic	NA	NA	NA	NA
20	Melospiza melodia	male reproductive success	Monomorphic	NA	NA	NA	NA
21	Larus michahellis	antioxidant defence, endocrine signal, & body mass	Dimorphic	yes	yes	yes	yes
22	Taeniopygia guttata	neural traits associated with song	Dimorphic	yes	yes (resources) no (sex)	yes (resources) no (sex)	no
23	Taeniopygia guttata	morphology, male sexual activity & basal metabolic rate	Dimorphic & Polymorphic	yes	yes	yes	no
24	Hippopodina iririkiensis	fitness	Polymorphic	yes	yes	yes	yes
25	Heliocidaris erythrogramma armigera	fertilization & hatching success	Dimorphic	yes	yes	yes	no

26	Gasterosteus aculeatus	survival & body size	Polymorphic	yes	yes	yes	no
27	Salmo salar	fitness	Dimorphic	yes	yes	no	yes
28	Salmo salar	morphology	Dimorphic	yes	yes	yes	no
29	Gasterosteus aculeatus	body size & shape	Dimorphic & Polymorphic	yes	yes	yes (environment) no (sex)	no
30	Lasius niger	cuticular hydrocarbons, fertility, & head size	Dimorphic	yes	no	no	yes
31	Gryllodes sigilatus	oxidative damage, antioxidant protection, reproductive effort, lifespan, & ageing	Dimorphic	yes	?	yes	no
32	Gryllodes sigillatus	female spermatophylax feeding behavior & amino acid composition	Monomorphic	NA	NA	NA	NA
33	Teleogryllus commodus	mate choice	Dimorphic	yes	yes	yes	no
34	Gnatocerus cornutus	mandable size & locomotor activity	Dimorphic	yes	no	no	yes
35	Sepsis punctum	development rate	Dimorphic & Polymorphic	yes	yes	yes	no
36	Drosophila melanogaster	macronutrient preferences	Dimorphic	yes	?	yes	no
37	Drosophila simulans	cuticular hydrocarbons	Dimorphic	yes	yes	no	yes
38	Drosophila yakuba and santomea	pigmentation	Dimorphic	yes	yes	no	yes
39	Drosophila melanogaster	egg-to-adult viability	Dimorphic	yes	yes	yes	no
40	Drosophila melanogaster	lifespan	Dimorphic	yes	yes	yes	no
41	Drosophila melanogaster	cuticular hydrocarbons	Dimorphic	yes	yes	yes	no
42	Drosophila serrata	mating preferences	Dimorphic	yes	yes	yes	no
43	Drosophila simulans	cuticular hydrocarbons	Dimorphic	yes	yes	yes	yes
44	Drosophila serrata	fitness	Dimorphic & Polymorphic	yes	yes	yes	yes
45	Drosophila serrata	contact pheromones	Dimorphic & Polymorphic	yes	yes	yes	no
46	Drosophila melanogaster	fecundity & copula duration	Monomorphic	NA	NA	NA	NA
47	Drosophila serrata	cuticular hydrocarbons	Monomorphic	NA	NA	NA	NA
48	Drosophila simulans	heat tolerance	Monomorphic	NA	NA	NA	NA
49	Drosophila serrata	cuticular hydrocarbons	Polymorphic	yes	yes	no	yes
50	Drosophila simulans	cuticular hydrocarbons	Polymorphic	yes	yes	yes	no
51	Melitaea cinxia	metabolic rates	Dimorphic	yes	yes	no	yes
52	Tenebrio molitor	immune response, melanisation, & morphology	Dimorphic & Polymorphic	yes	?	yes	no

53	Tribolium castaneum	external immune defences	Monomorphic	NA	NA	NA	NA
54	Tribolium castaneum	growth & life history	Polymorphic	yes	yes	yes	no
55	Gryllus firmus	melanization, ovary/testis mass & DLM mass	Dimorphic & Polymorphic	yes	yes	yes	yes
56	Callosobruchus maculatus	fitness	Dimorphic	yes	no	yes	no
57	Nauphoeta cinerea	discontinuous gas exchange, metabolic rate, & body mass	Dimorphic	yes	yes	no	yes
58	Enchenopa binotata	female mate preference	Monomorphic	NA	NA	NA	NA
59	Enchenopa binotata	mate preferences	Monomorphic	NA	NA	NA	NA
60	Bemisia tabaci	survival & fecundity	Dimorphic	yes	yes	no	yes
61	Bos taurus	social dominance	Monomorphic	NA	NA	NA	NA
62	Homo sapiens	ages at first & last reproduction	Dimorphic	yes	?	yes	yes
63	Homo sapiens	metabolic profiles	Monomorphic	NA	NA	NA	NA
64	laboratory house mice	wheel running behavior	Dimorphic	yes	yes	yes	yes
65	Suricata suricatta	growth & size	Dimorphic	yes	yes	yes	yes
66	Ovis aries	antibody titer & body weight	Polymorphic	yes	yes	yes	no
67	Saimiri boliviensis	neonate mass	Dimorphic	yes	yes	yes	no
68	Ipomoea purpurea	fitness & tolerance to competition	Dimorphic	yes	yes	yes	no
69	Plantago coronopus	reproductive & vegetative traits	Dimorphic & Polymorphic	yes	yes	yes	no
70	Solanum carolinense	resistance to herbivory	Monomorphic	NA	NA	NA	NA
71	Pinus pinaster	early seedling performance	Dimorphic	yes	yes	yes	no
72	Ipomoea hederacea	size, flowering time, & seed number	Monomorphic	NA	NA	NA	NA
73	Arabidopsis lyrata	seed size	Monomorphic	NA	NA	NA	NA
74	Geranium carolinianum	competitive ability	Polymorphic	yes	no	yes	no
75	Arabidopsis lyrata	plant performance & coping with drought	Polymorphic	yes	yes	no	yes
76	Arabidopsis thaliana	13 varied traits	Polymorphic	yes	yes	yes	yes
77	Pinus radiata	resistance to herbivores	Dimorphic	yes	yes	yes	no
78	Eucalyptus globulus	disease resistance	Polymorphic	yes	yes	yes	no
79	Schistocephalus solidus	fitness	Monomorphic	NA	NA	NA	NA

Literature Cited for Table S2.1

1. Welch, A. M., Smith, M. J., & Gerhardt, H. C. (2014). A multivariate analysis of genetic variation in the advertisement call of the gray treefrog, Hyla versicolor. *Evolution*, *68*(6), 1629–39. doi:10.1111/evo.12397
2. Gomez-Mestre, I., & Warkentin, K. M. (2013). Risk-induced hatching timing shows low heritability and evolves independently of spontaneous hatching in red-eyed treefrogs. *Journal of Evolutionary Biology*, *26*(5), 1079–1089. doi:10.1111/jeb.12121
3. Steinsland, I., Larsen, C. T., Roulin, A., & Jensen, H. (2014). Quantitative genetic modeling and inference in the presence of nonignorable missing data. *Evolution*, *68*(6), 1735–1747. doi:10.1111/evo.12380
4. Dreiss, A.N., Ruppli, C.A., & Roulin, A. (2013). Individual vocal signatures in barn owl nestlings: does individual recognition have an adaptive role in sibling vocal competition? *Journal of Evolutionary Biology*, *27*, 63–75. doi:10.1111/jeb.12277
5. Møller, A. P. (2014). Life history, predation and flight initiation distance in a migratory bird. *Journal of Evolutionary Biology*, *27*(6), 1105–1113. doi:10.1111/jeb.12399
6. Hadfield, J. D., Heap, E.A., Bayer, F., Mittell, E.A., & Crouch, N.M.A. (2013b). Intraclutch differences in egg characteristics mitigate the consequences of age-related hierarchies in a wild passerine. *Evolution*, *67*(9), 2688–2700. doi:10.1111/evo.12143
7. Hadfield, J. D., Heap, E.A., Bayer, F., Mittell, E.A., & Crouch, N. M.A. (2013a). Disentangling genetic and prenatal sources of familial resemblance across ontogeny in a wild passerine. *Evolution*, *67*(9), 2701–2713. doi:10.1111/evo.12144
8. Bjorklund, M., Husby, A., & Gustafsson, L. (2013). Rapid and unpredictable changes of the G-matrix in a natural bird population over 25 years. *Journal of Evolutionary Biology*, *26*, 1–13. doi:10.1111/jeb.12044
9. Husby, A., Schielzeth, H., Forstmeier, W., Gustafsson, L., & Qvarnstrom, A. (2013). Sex chromosome linked genetic variance and the evolution of sexual dimorphism of quantitative traits. *Evolution*, *67*(3), 609–619. doi:10.5061/dryad.3hq60
10. Kim, S.-Y., Fargallo, J. A., Vergara, P., & Martinez-Padilla, J. (2013). Multivariate heredity of melanin-based coloration, body mass and immunity. *Heredity*, *111*(2), 139–146. doi:10.1038/hdy.2013.29
11. Tarka, M., Akesson, M., Hasselquist, D., & Hansson, B. (2014). Intralocus sexual conflict over wing length in a wild migratory bird. *The American Naturalist*, *183*(1), 62–73. doi:10.1086/674072
12. Losdat, S., Helfenstein, F., Blount, J. D., & Richner, H. (2014). Resistance to oxidative stress shows low heritability and high common environmental variance in a wild bird. *Journal of Evolutionary Biology*, *27*, 1990–2000. doi:10.1111/jeb.12454
13. Nicolaus, M., Brommer, J. E., Ubels, R., Tinbergen, J. M., & Dingemanse, N. J. (2013). Exploring patterns of variation in clutch size-density reaction norms in a wild passerine bird. *Journal of Evolutionary Biology*, *26*(9), 2031–2043. doi:10.1111/jeb.12210
14. Schaper, S. V, Gienapp, P., Dawson, A., & Visser, M. E. (2013). Heritability of gonad size varies across season in a wild songbird. *Journal of Evolutionary Biology*, *26*(12), 2739–2745. doi:10.1111/jeb.12249

15. Gienapp, P., van Noordwijk, A.J., & Visser, M. E. (2013). Genetic background, and not ontogenetic effects, affects avian seasonal timing of reproduction. *Journal of Evolutionary Biology*, *26*(10), 2147–2153. doi:10.1111/jeb.12205

16. Evans, S. R., Schielzeth, H., Forstmeier, W., Sheldon, B. C., & Husby, A. (2014). Nonautosomal genetic variation in carotenoid coloration. *The American Naturalist*, *184*(3), 374–383. doi:10.1086/677397

17. Weiß, B. M., & Foerster, K. (2013). Age and sex affect quantitative genetic parameters for dominance rank and aggression in free-living greylag geese. *Journal of Evolutionary Biology*, *26*(2), 299–310. doi:10.1111/jeb.12042

18. Gienapp, P., & Merilä, J. (2014). Disentangling plastic and genetic changes in body mass of Siberian jays. *Journal of Evolutionary Biology*, *27*, 1849–1858. doi:10.1111/jeb.12438

19. Reid, J. M., Arcese, P., Keller, L. F., & Losdat, S. (2014). Female and male genetic effects on offspring paternity: additive genetic (co)variances in female extra-pair reproduction and male paternity success in song sparrows (Melospiza melodia). *Evolution*, *68*(8), 2357–2370. doi:10.1111/evo.12424

20. Reid, J. M., Arcese, P., & Losdat, S. (2014). Genetic covariance between components of male reproductive success: within-pair vs. extra-pair paternity in song sparrows. *Journal of Evolutionary Biology*, *27*(10), 2046–56. doi:10.1111/jeb.12445

21. Kim, S.-Y., Noguera, J. C., Tato, A., & Velando, A. (2013). Vitamins, stress and growth: the availability of antioxidants in early life influences the expression of cryptic genetic variation. *Journal of Evolutionary Biology*, *26*(6), 1341–1352. doi:10.1111/jeb.12136

22. Woodgate, J. L., Buchanan, K. L., Bennett, A. T. D., Catchpole, C. K., Brighton, R., & Leitner, S. (2014). Environmental and genetic control of brain and song structure in the zebra finch. *Evolution*, *68*(1), 230–240. doi:10.1111/evo.12261

23. Mathot, K. J., Martin, K., Kempenaers, B., & Forstmeier, W. (2013). Basal metabolic rate can evolve independently of morphological and behavioural traits. *Heredity*, *111*(3), 175–81. doi:10.1038/hdy.2013.35

24. Monro, K., & Marshall, D. J. (2013). Evolutionary constraints and the maintenance of individual specialization throughout succession. *Evolution*, *67*(12), 3636–3644. doi:10.1111/evo.12220

25. Lymbery, R. a, & Evans, J. P. (2013). Genetic variation underlies temperature tolerance of embryos in the sea urchin Heliocidaris erythrogramma armigera. *Journal of Evolutionary Biology*, *26*(10), 2271–2282. doi:10.1111/jeb.12225

26. DeFaveri, J., & Merilä, J. (2014). Local adaptation to salinity in the three-spined stickleback? *Journal of Evolutionary Biology*, *27*(2), 290–302. doi:10.1111/jeb.12289

27. Houde, A.L., Wilson, C. C., & Neff, B. D. (2013). Genetic architecture of survival and fitness-related traits in two populations of Atlantic salmon. *Heredity*, *111*(6), 513–9. doi:10.1038/hdy.2013.74

28. Debes, P. V, Fraser, D. J., McBride, M. C., & Hutchings, J.A. (2013). Multigenerational hybridisation and its consequences for maternal effects in Atlantic salmon. *Heredity*, *111*(3), 238–47. doi:10.1038/hdy.2013.43

29. Ramler, D., Mitteroecker, P., Shama, L. N. S., Wegner, K. M., & Ahnelt, H. (2014). Nonlinear effects of temperature on body form and developmental canalization in the threespine stickleback. *Journal of Evolutionary Biology*, *27*, 497–507. doi:10.1111/jeb.12311

30. Holman, L., Linksvayer, T. A., & D'Ettorre, P. (2013). Genetic constraints on dishonesty and caste dimorphism in an ant. *The American Naturalist*, *181*(2), 161–170. doi:10.1086/668828

31. Archer, C. R., Sakaluk, S. K., Selman, C., Royle, N. J., & Hunt, J. (2013). Oxidative stress and the evolution of sex differences in life span and ageing in the decorated cricket, Gryllodes sigillatus. *Evolution*, *67*(3), 620–634. doi:10.5061/dryad.cs16k

32. Gershman, S. N., Hunt, J., & Sakaluk, S. K. (2013). Food fight: sexual conflict over free amino acids in the nuptial gifts of male decorated crickets. *Journal of Evolutionary Biology*, *26*(4), 693–704. doi:10.1111/jeb.12078

33. Hall, M. D., Lailvaux, S. P., & Brooks, R. C. (2013). Sex-specific evolutionary potential of pre- and postcopulatory reproductive interactions in the field cricket, Teleogryllus commodus. *Evolution*, *67*(6), 1831–1837. doi:10.1111/evo.12067

34. Fuchikawa, T., & Okada, K. (2013). Inter- and intrasexual genetic correlations of exaggerated traits and locomotor activity. *Journal of Evolutionary Biology*, *26*(9), 1979–1987. doi:10.1111/jeb.12197

35. Berger, D., Postma, E., Blanckenhorn, W. U., & Walters, R. J. (2013). Quantitative genetic divergence and standing genetic (co)variance in thermal reaction norms along latitude. *Evolution*, *67*(8), 2385–2399. doi:10.1111/evo.12138

36. Reddiex, A. J., Gosden, T. P., Bonduriansky, R., & Chenoweth, S. F. (2013). Sex-specific fitness consequences of nutrient intake and the evolvability of diet preferences. *The American Naturalist*, *182*(1), 91–102. doi:10.1086/670649

37. Ingleby, F. C., Hunt, J., & Hosken, D. J. (2013). Heritability of male attractiveness persists despite evidence for unreliable sexual signals in Drosophila simulans. *Journal of Evolutionary Biology*, *26*(2), 311–324. doi:10.1111/jeb.12045

38. Matute, D. R., & Harris, A. (2013). The influence of abdominal pigmentation on desiccation and ultraviolet resistance in two species of Drosophila. *Evolution*, *67*(8), 2451–2460. doi:10.1111/evo.12122

39. Ketola, T., Kellermann, V. M., Loeschcke, V., López-Sepulcre, A., & Kristensen, T. N. (2014). Does environmental robustness play a role in fluctuating environments? *Evolution*, *68*(2), 587–594. doi:10.1111/evo.12285

40. Lehtovaara, A., Schielzeth, H., Flis, I., & Friberg, U. (2013). Heritability of life span is largely sex limited in Drosophila. *The American Naturalist*, *182*(5), 653–665. doi:10.1086/673296

41. Ingleby, F. C., Innocenti, P., Rundle, H. D., & Morrow, E. H. (2014). Between-sex genetic covariance constrains the evolution of sexual dimorphism in Drosophila melanogaster. *Journal of Evolutionary Biology*, *27*(8), 1721–1732. doi:10.1111/jeb.12429

42. Gosden, T. P., & Chenoweth, S. F. (2014). The evolutionary stability of cross-sex, cross-trait genetic covariances. *Evolution*, *68*(6), 1687–1697. doi:10.1111/evo.12398

43. Ingleby, F. C., Hosken, D. J., Flowers, K., Hawkes, M. F., Lane, S. M., Rapkin, J., Dworkin, I., & Hunt, J. (2013). Genotype-by-environment interactions for cuticular hydrocarbon expression in Drosophila simulans. *Journal of Evolutionary Biology*, *26*(1), 94–107. doi:10.1111/jeb.12030

44. Punzalan, D., Delcourt, M., & Rundle, H. D. (2014). Comparing the intersex genetic correlation for fitness across novel environments in the fruit fly, Drosophila serrata. *Heredity*, *112*(2), 143–148. doi:10.1038/hdy.2013.85

45. Gosden, T. P., Rundle, H. D., & Chenoweth, S. F. (2014). Testing the correlated response hypothesis for the evolution and maintenance of male mating preferences in Drosophila serrata. *Journal of Evolutionary Biology*, *27*(10), 2106–12. doi:10.1111/jeb.12461

46. Edward, D. A, Poissant, J., Wilson, A. J., & Chapman, T. (2014). Sexual conflict and interacting phenotypes: a quantitative genetic analysis of fecundity and copula duration in Drosophila melanogaster. *Evolution*, *68*(6), 1651–1660. doi:10.1111/evo.12376

47. Hine, E., McGuigan, K., & Blows, M. W. (2014). Evolutionary constraints in high-dimensional trait sets. *The American Naturalist*, *184*(1), 119–131. doi:10.1086/676504

48. Van Heerwaarden, B., & Sgrò, C. M. (2013). Multivariate analysis of adaptive capacity for upper thermal limits in Drosophila simulans. *Journal of Evolutionary Biology*, *26*(4), 800–809. doi:10.1111/jeb.12090

49. Aguirre, J. D., Hine, E., Mcguigan, K., & Blows, M. W. (2014). Comparing G : multivariate analysis of genetic variation in multiple populations. *Heredity*, *112*(1), 21–29. doi:10.1038/hdy.2013.12

50. Ingleby, F. C., Hosken, D. J., Flowers, K., Hawkes, M. F., Lane, S. M., Rapkin, J., House, C. M., Sharma, M. D., & Hunt, J. (2014). Environmental heterogeneity, multivariate sexual selection and genetic constraints on cuticular hydrocarbons in Drosophila simulans. *Journal of Evolutionary Biology*, *27*(4), 700–713. doi:10.1111/jeb.12338

51. Mattila, a L. K., & Hanski, I. (2014). Heritability of flight and resting metabolic rates in the Glanville fritillary butterfly. *Journal of Evolutionary Biology*, *27*(8), 1733–1743. doi:10.1111/jeb.12426

52. Prokkola, J., Roff, D., Kärkkäinen, T., Krams, I., & Rantala, M. J. (2013). Genetic and phenotypic relationships between immune defense, melanism and life-history traits at different temperatures and sexes in Tenebrio molitor. *Heredity*, *111*(2), 89–96. doi:10.1038/hdy.2013.20

53. Joop, G., Roth, O., Schmid-Hempel, P., & Kurtz, J. (2014). Experimental evolution of external immune defences in the red flour beetle. *Journal of Evolutionary Biology*, *27*(8), 1562–1571. doi:10.1111/jeb.12406

54. Irwin, K. K., & Carter, P. A. (2013). Constraints on the evolution of function-valued traits: a study of growth in Tribolium castaneum. *Journal of Evolutionary Biology*, *26*(12), 2633–2643. doi:10.1111/jeb.12257

55. Roff, D. A., & Fairbairn, D. J. (2013). The costs of being dark: the genetic basis of melanism and its association with fitness-related traits in the sand cricket. *Journal of Evolutionary Biology*, *26*(7), 1406–1416. doi:10.1111/jeb.12150

56. Berger, D., Grieshop, K., Lind, M. I., Goenaga, J., Maklakov, A. A., & Arnqvist, G. (2014). Intralocus sexual conflict and environmental stress. *Evolution*, *68*(8), 2184–2196. doi:10.1111/evo.12439

57. Schimpf, N. G., Matthews, P. G. D., & White, C. R. (2013). Discontinuous gas exchange exhibition is a heritable trait in speckled cockroaches Nauphoeta cinerea. *Journal of Evolutionary Biology*, *26*(7), 158815–97. doi:10.1111/jeb.12093

58. Rodríguez, R. L., Hallett, A. C., Kilmer, J. T., & Fowler-Finn, K. D. (2013). Curves as traits: genetic and environmental variation in mate preference functions. *Journal of Evolutionary Biology*, *26*(2), 434–442. doi:10.1111/jeb.12061

59. Rebar, D., & Rodríguez, R. L. (2014). Genetic variation in host plants influences the mate preferences of a plant-feeding insect. *The American Naturalist*, *184*(4), 489–99. doi:10.1086/677751

60. Díaz, F., Muñoz-Valencia, V., Juvinao-Quintero, D. L., Manzano-Martínez, M. R., Toro-Perea, N., Cárdenas-Henao, H., & Hoffmann, A. A. (2014). Evidence for adaptive divergence of thermal responses among Bemisia tabaci populations from tropical Colombia following a recent invasion. *Journal of Evolutionary Biology*, *27*(6), 1160–1171. doi:10.1111/jeb.12387

61. Sartori, C., & Mantovani, R. (2013). Indirect genetic effects and the genetic bases of social dominance : evidence from cattle. *Heredity*, *110*(1), 3–9. doi:10.1038/hdy.2012.56

62. Bürkli, A., & Postma, E. (2014). Genetic constraints underlying human reproductive timing in a premodern Swiss village. *Evolution*, *68*(2), 526–537. doi:10.1111/evo.12287

63. Alul, F. Y., Cook, D. E., Shchelochkov, O. A., Fleener, L. G., Berberich, S. L., Murray, J. C., & Ryckman, K. K. (2013). The heritability of metabolic profiles in newborn twins. *Heredity*, *110*(3), 253–8. doi:10.1038/hdy.2012.75

64. Careau, V., Wolak, M. E., Carter, P. A., & Garland, T. J. (2013). Limits to behavioral evolution: the quantitative genetics of a complex trait under directional selection. *Evolution*, *67*(11), 3102–19. doi:10.1111/evo.12200

65. Huchard, E., Charmantier, A., English, S., Bateman, A., Nielsen, J. F., & Clutton-Brock, T. (2014). Additive genetic variance and developmental plasticity in growth trajectories in a wild cooperative mammal. *Journal of Evolutionary Biology*, *27*(9), 1893–1904. doi:10.1111/jeb.12440

66. Hayward, A. D., Garnier, R., Watt, K. A., Pilkington, J. G., Grenfell, B. T., Matthews, J. B., Pemberton, J.M., Nussey, D. H., & Graham, A. L. (2014). Heritable, heterogeneous, and costly resistance of sheep against nematodes and potential feedbacks to epidemiological dynamics. *The American Naturalist*, *184*(Suppl), S58–76. doi:10.1086/676929

67. Blomquist, G. E., & Williams, L. E. (2013). Quantitative genetics of costly neonatal sexual size dimorphism in squirrel monkeys (Saimiri boliviensis). *Journal of Evolutionary Biology*, *26*(4), 756–765. doi:10.1111/jeb.12096

68. Chaney, L., & Baucom, R. S. (2014). The costs and benefits of tolerance to competition in ipomoea purpurea, the common morning glory. *Evolution*, *68*(6), 1698–16709. doi:10.1111/evo.12383

69. Hansen, C. F., García, M. B., & Ehlers, B. K. (2013). Water availability and population origin affect the expression of the tradeoff between reproduction and growth in Plantago coronopus. *Journal of Evolutionary Biology*, *26*(5), 993–1002. doi:10.1111/jeb.12114

70. Wise, M. J., & Rausher, M. D. (2013). Evolution of resistance to a multiple-herbivore community: genetic correlations, diffuse coevolution, and constraints on the plant's response to selection. *Evolution*, *67*(6), 1767–1779. doi:10.1111/evo.12061

71. Zas, R., Cendán, C., & Sampedro, L. (2013). Mediation of seed provisioning in the transmission of environmental maternal effects in Maritime pine (Pinus pinaster Aiton). *Heredity*, *111*(3), 248–55. doi:10.1038/hdy.2013.44

72. Stinchcombe, J. R., Simonsen, A. K., & Blows, M. W. (2013). Estimating uncertainty in multivariate responses to selection. *Evolution*, *68*(4), 1188–1196. doi:10.1111/evo.12321

73. Willi, Y. (2013). The battle of the sexes over seed size: support for both kinship genomic imprinting and interlocus contest evolution. *The American Naturalist*, *181*(6), 787–798. doi:10.1086/670196

74. Shirk, R. Y., & Hamrick, J. L. (2014). Multivariate adaptation but no increase in competitive ability in invasive geranium carolinianum L. (Geraniaceae). *Evolution*, *68*(10), 2945–59. doi:10.1111/evo.12474

75. Paccard, A., Vance, M., & Willi, Y. (2013). Weak impact of fine-scale landscape heterogeneity on evolutionary potential in Arabidopsis lyrata. *Journal of Evolutionary Biology*, *26*(11), 2331–2340. doi:10.1111/jeb.12220

76. Tonsor, S. J., Elnaccash, T. W., & Scheiner, S. M. (2013). Developmental instability is genetically correlated with phenotypic plasticity, constraining heritability, and fitness. *Evolution*, *67*(10), 2923–2935. doi:10.1111/evo.12175

77. Moreira, X., Zas, R., & Sampedro, L. (2013). Additive genetic variation in resistance traits of an exotic pine species: little evidence for constraints on evolution of resistance against native herbivores. *Heredity*, *110*(5), 449–56. doi:10.1038/hdy.2012.108

78. Hamilton, M. G., Williams, D. R., Tilyard, P. A., Pinkard, E. A., Wardlaw, T. J., Glen, M., Vaillancourt, M. E., & Potts, B. M. (2013). A latitudinal cline in disease resistance of a host tree. *Heredity*, *110*(4), 372–9. doi:10.1038/hdy.2012.106

79. Benesh, D. P. (2013). Parental effects on the larval performance of a tapeworm in its copepod first host. *Journal of Evolutionary Biology*, *26*(8), 1625–1633. doi:10.1111/jeb.12165
